# The uneven triad: a qualitative study of perspectives of relatives, patients, and professionals on (not) involving relatives in mental health

**DOI:** 10.1186/s12888-025-06814-3

**Published:** 2025-04-16

**Authors:** Suzanne J.C. Kroon, Lian van der Krieke, Richard Bruggeman, Manna A. Alma

**Affiliations:** 1 Department of Psychiatry, Rob Giel Research Center, University of Groningen, University Medical Center Groningen, P.O. box 30.001, Groningen, 9700 RB The Netherlands; 2 GGZ Drenthe, Hoogeveen, The Netherlands; 3 Department of Health Sciences, Applied Health Research, University Medical Center Groningen, University of Groningen, Groningen, The Netherlands

**Keywords:** Involvement of relatives, Perspectives of relatives, Patients and professionals, Collaboration in the triad, Qualitative study

## Abstract

**Introduction:**

Recognition of the value of involving relatives in mental health care is growing. This study explores the ambivalence surrounding their role, including formalisation, instrumental use, burden, and potential benefits. Collaboration between relatives, patients, and professionals is essential to recovery-based approaches. Thus, despite challenges, the involvement of relatives remains crucial. This study aims to understand perspectives on involving relatives in mental health care, within the larger context of collaboration across the triad of relatives, patients, and professionals.

**Methods:**

We conducted a qualitative study using semi-structured interviews with relatives (*n* = 7), patients (*n* = 7), and professionals (*n* = 10) connected to various mental health care organizations. The study took place in The Netherlands. Data were analysed using thematic analysis.

**Results:**

For collaboration in the triad, we found five aspects to be of importance: the significance of involving relatives, changing roles from relative to caregiver, relatives’ intermediary role in patient-professional relationship, negative experiences of relatives in the triad, and ambivalence about patient’s autonomy. Notably, because collaboration between professionals and relatives is often challenging, it often leads to tensions.

**Conclusion:**

Our study uncovers varying perspectives both on involving relatives and on collaboration within the triad. Recovery-oriented approaches do not always align with patients’ and relatives’ intentions and values. Although relatives want to be involved, they often feel unheard and unseen by professionals. Relatives’ roles -especially the intermediary role- are surprisingly underrepresented in the recovery-oriented literature. This study reveals several tensions between the three perspectives, culminating in a so-called uneven triad. We conclude that although the concept of involving relatives is highly advocated in recovery-based approaches, actual practice is lagging behind.

## Introduction

### Relatives

Recent years have seen a growing acknowledgement by scholars and health care professionals of the significance of involving relatives in mental care for people with severe mental illnesses (SMI) [1]. However, as their role is not unequivocal, multiple perspectives need to be studied. Relatives often serve as informal caregivers, taking on various roles while caring for their families. As defined by Zarzycky & Morrison: “*Informal caregivers are those providing care*,* which exceeds that which is typically provided*,* to a relative or friend with care needs*” [2].

The model of caregiving roles by Twigg is still useful for exploring the different roles played by caregivers in the lives of care receivers [3, 4]. A relative can be a resource, co-worker, co-client, or care coordinator. As ‘resources’, relatives -significant others, not only family-members but also friends- are crucial, long before a person comes into care. Relatives often remain involved as ‘co-workers’ because they continue to care for their family members. As part of an informal network, relatives often have extra tasks, such as attending consultations with professionals to discuss goals set by their family members and whether these goals have been achieved. On the one hand, stress caused by their family member’s situation can turn relatives into ‘(co)patients’ themselves. On the other hand, relatives who take up a role as care ‘coordinators’ may themselves almost become professionals. Overall, relatives offer company, give emotional support, and offer help with various practical matters [5, 6].

The responsibilities of a family caregiver may be crucial, but they are also demanding [7, 8]. Relatives expressing a desire to assist, may find their lives dedicated to serving a person with mental health challenges [9, 10]. Such support can necessitate adjustments to their daily routines, such as potential reductions in working hours or even cessation of work altogether. Caregivers must, therefore, seriously consider their own well-being [4, 5, 11]. In certain cases, the care of a family member can become too demanding, resulting in the development of (mental) health issues for the caregiver themselves. The concept of the “burden of relatives” is a well-established one, and this is not without cause [12]. They may also face a risk of disrupting the relationship with their son, daughter, friend, or partner who has mental health challenges. This risk, characterised in terms of “ambivalent relationships”, can challenge the balance between one’s role as a caregiver and as a family member [1, 6]. Nevertheless, in their role as caregivers, family members can also derive positive effects, such as a sense of purpose and the satisfaction of contributing to something meaningful [13].

### Recovery-oriented care

In contrast to the traditional medical model, the recovery model is a more holistic, person-centred mental health care approach, which has quickly gained momentum and is becoming the standard model of mental health care [14]. In this model, the patient has autonomy and is in control, involving relatives in the recovery process; the professionals are not in the lead but have a more supporting role [15]. Family, friends, or other relatives constitute the informal network of the client, whereas professionals constitute the formal network within the resource group or support group [16, 17]. Recovery-based care focuses not only on clinical symptoms but explicitly addresses social and personal recovery [18, 19]. The overarching aim of the recovery model is to empower individuals to lead fulfilling lives by fostering hope and the achievement of personal goals, promoting social quality, and nurturing supportive contacts [20, 21].

### Collaboration

As emphasised by various scholars, when involving relatives, collaboration is key [11, 22]. The present study focuses on interpersonal collaboration among relatives, patients, and professionals, commonly called the ‘(therapeutic) triad’. Ideally, in recovery-based approaches, professionals do interact with patients but at the same time actively engage with their relatives. However, in the care of family members with SMI this practice can lead to ambivalence, characterised by the formalisation of family roles, the instrumental use of relatives, and the potential benefits and burdens associated with their involvement [23, 24]. Indeed, one can argue that consideration for the worries and feelings of relatives in the triad is limited, and that acknowledging their impact and addressing their concerns can be challenging [25–28].

### Research question

Although in recovery-based approaches the importance of involving relatives is undisputed, it has not yet become standard practice. This study will explore the perspectives of relatives, patients and professionals on the involvement of relatives in recovery-oriented treatment. Our research question therefore is: How do relatives themselves, patients and professionals perceive the involvement of relatives in recovery-oriented treatment? To our knowledge, this study is the one of the first to combine all three perspectives on involving relatives in mental health care and collaboration. By doing so, we hope to shed more light on the collaborative aspect of patients, relatives, and professionals working together in the triad.

## Methods

### Design and setting

The interpretivism paradigm is employed, given that, by definition, the experiences of the participants are subjective. The study is underpinned by a phenomenological approach with interest focused on the experiences of the participants while working together in the triad. Using thematic analysis, this qualitative interview study regarding the involvement of relatives in recovery-based approaches focused on three perspectives: those of relatives, patients, and professionals. The patients and professionals were connected to various mental health organisations (*n* = 6) in the province of Groningen, in the North of the Netherlands.

Our study was part of a larger study called FOCUS, which studies the social integration of people with psychosis, and methods of effective collaboration in community care and supported housing. To set up the methods section we used the COREQ checklist [29].

### Ethical considerations

The Medical Ethics Review Board of the University Medical Center Groningen approved the FOCUS study with reference number 201,900,607. Written informed consent was obtained from all participants.

### Participants

Our study included relatives, patients, and professionals. These participants were not related to each other and therefore we could not interview complete triads, for example son, father, psychiatrist from one triad. Relatives were included if they participated in a resource or support group or if they were otherwise connected to their son, daughter, friend, sibling, partner, or neighbour within a recovery-oriented context including those with family experience expertise. For patients, inclusion criteria were: (1) receiving treatment from a mental health care organisation; (2) being currently (or within the past six months) engaged in a recovery-oriented approach, and (3) residing in the province of Groningen. Professionals were eligible for participation if they were involved in recovery-oriented approaches that included relatives.

For our study we started with organisations from the Program for Mentally Vulnerable persons (PMV), and later also included other organisations. The mental health care organisations involved have similar kinds of patients with SMI and used a recovery-based approach, which includes the involvement of relatives. The PMV, which is a regional project, aimed at enhancing mutual understanding between various regional organisations in their collaborating on addressing the well-being of mentally vulnerable persons [30]. The professionals were selected by the organizations involved; these organizations were asked to nominate a professional involved in recovery-oriented care. The search for relatives and patients was initiated with the help of professionals, who were used as gatekeepers, including specialist mental health nurses, who were instructed to request participation from a diverse sample of patients and relatives. This instruction included an extensive email with an attached information letter and informed consent form for those who may want to participate in the study. This approach did not generate a sufficient number of participants; only four were recruited. This was insufficient to reach data saturation. Then, through snowball sampling, each interviewed professional was asked to recommend one additional person (patient or family member) for the study. This process may have led to the exclusion of patients who did not have a family/social network. The interviewed professionals were selected by the organizations involved, with the obvious requirement that they meet the inclusion criteria. One patient who had promised to be interviewed eventually found it too stressful/burdensome and cancelled the interview. In this study when referring to relatives as ‘caregivers’ we primarily mean parents, partners, and siblings.

### Data collection

All interviews were conducted by SK (MSc, PhD student, and at that time, senior policy adviser at a municipality, female (F)) between November 2021 and May 2023. At the start of each interview, the researcher informed the interviewee about [1] the goal of the research [2], the interview topics and [3] the opportunity for interviewees to receive a summary of the interview as a form of member checking. If they had any comments, they could adjust them to the text. SK is a trained interviewer.

We used distinct semi-structured interview guides. Interviews with relatives focused on their experiences within support groups, their sense of being seen and heard, their needs, and the extent to which their care contributed to the recovery of their child or partner. Interviews with patients focused on their experiences in collaborating with professionals, relatives, formal and informal networks; and their own personal journeys. Professionals’ topics included recovery-based approaches, ways of involving relatives in mental health care, formal and informal networks, and interrelationships among mental health organisations.

Interviews lasted between 45 and 75 min with only interviewee and researcher present. Ten interviews were conducted via Microsoft Teams; fourteen were conducted live at the interviewee’s workplace or home, or the researcher’s workplace. Interviews with professionals were conducted during the COVID-19 pandemic and were therefore conducted online. Subsequent to the conclusion of the Dutch corona pandemic, further interviews were conducted with patients and relatives. On occasion, a patient or relatives opted for an online interview, and these were conducted with even greater care to obtain a comprehensive “rapport”. The majority of patients were already engaged in long-term care, and relatives were also involved for an extended period. Consequently, we contend that the experiences documented in this study are not solely confined to the period surrounding the pandemic but extend to the context of regular times as well. All interviews were audio-recorded and transcribed verbatim. Along with the interviews, we also used a reflexive logbook to bracket and record notable aspects [31]. We collected data until saturation was reached, and then discussed them with the research team.

### Data analysis

Thematic analysis [32]was utilized for the analysis of the data, a process comprised of six stages which are not always linear, but sometimes cyclical. Initially, SK perused all transcripts and documented the initial themes, such as the significance of relatives (step 1). In the subsequent phase, two researchers, SK and MA (PhD, social scientist, F), used the software program Atlas.ti 23 to code the data using a cyclical and inductive process. New codes emerged during the coding process, and coherent codes were subsequently grouped into category codes, such as roles. The final code tree comprised 90 codes and 11 category codes (step 2). Thirdly, following the coding stage, SK and MA further classified the codes into initial themes (step 3). These themes were then discussed with other members of the research team, namely LvdK (PhD, researcher and healthcare psychologist, F) and RB (MD, PhD, professor and psychiatrist, male (M)). The themes underwent a process of refinement based on these discussions (step 4). The themes were then redefined on multiple occasions. After consensus on the themes, quotations were selected to illustrate the themes (step 5). The data were validated through a review of the research questions and relevant literature (step 6). The quotations were translated into English by a native speaker.

### Reflexivity

To gain insight into the assumptions underlying the research topic, the first author (SK) maintained a reflexive logbook. One topic in the logbook was her personal experience as a caregiver for a family member, which enabled her to identify with the struggles faced by the relatives. Although she did not disclose her own experiences to the interviewees, this background fostered a deeper motivation to highlight their narratives. During research team meetings, the researchers discussed strategies for managing this connection, which facilitated empathy in the interviews, without allowing personal experiences to overshadow the process. These positionality meetings were regularly conducted to systematically examine each team member’s relationship to the research topic. Various methods were employed to ensure and maintain a fresh and critical perspective regarding our actions as qualitative researchers [33].

At the time of the study, as a senior policy adviser for the municipality of Groningen, SK knew some of the interviewees personally. To prevent prejudice and bias, she used bracketing [31]. Writing down all she knew about the interviewees and discussing it with her supervisors enabled her to attend the interviews as open-minded as possible.

## Results

We enrolled a total of 24 participants in this study (see also Table 1); 7 were relatives, 7 were patients, and 10 were professionals. The relatives (3 male/4 female) were aged between 46 and 70; the patients (2 male/5 female) between 31 and 64; and the professionals (4 male/6 female) between 34 and 51. The professionals included team managers in mental health care, psychiatric nurses, and case managers. They were responsible for managing, securing, and implementing methods that involved relatives in treatment. The patients and relatives came from diverse backgrounds, ranging from highly educated to less educated. The patients and relatives lived in a city or in a village next to this city. Substance abuse was not an exclusion criteria, however, the interviews did not reveal any cases of substance abuse. The professionals interviewed were a more homogeneous group, largely highly educated. No data was collected about professionals’ religion or place of residence.

**Table 1:** Characteristics of participants

Relative nr. (*R*)/ Patient nr. (*P*)/ Health care Professional nr. (H)	Gender Female (F) /Male (M)	Age in years	Relationship relatives towards patients
Relatives			
R1	F	61	Partner
R2	F	46	Partner
R3	F	66	Mother
R4	F	58	Partner/Mother/Sister
R5	M	70	Father
R6	M	70	Father
R7	M	59	Partner
Patients			
P1	M	39	-
P2	F	37	
P3	F	64	
P4	F	38	
P5	M	56	
P6	F	32	
P7	F	31	
Health care professionals			
H 1	M	34	-
H 2	F	35	
H 3	F	36	
H 4	M	51	
H 5	F	50	
H 6	F	40	
H 7	M	40	
H 8	M	46	
H 9	F	37	
H 10	F	41	

Collaboration in the triad takes place between relatives, patients, and professionals. We found four major themes: the significance of involving relatives, changing roles from relative to caregiver, relatives’ intermediary role in patient-professional relationship, and negative experiences of relatives in the triad. One further, minor, theme was ambivalence about patient’s autonomy. All themes had their effect on collaboration in the triad.

### Themes

#### Significance of involving relatives

All participants agreed that the involvement of relatives is one of the most important factors in a patient’s recovery. Patients indicated that their relatives supported them in several ways: relatives are there when patients need them, offering stability; relatives also comfort and think along with patients. This makes patients feel heard and seen.

P5: *“So*,* when my parents were there for me in that hard time*,* they gave me a lot of shelter and support.”*

Patients noted that the involvement of relatives made them feel calmer and more confident, and able to leave their homes again. Most patients also mentioned that sharing their feelings and thoughts with relatives made it easier for them to deal with their mental state. All patients said they were satisfied with the collaboration with their relatives. Even those who had previously said they did not want their relatives collaborating in the triad, were positive about their involvement. This applied both to their relatives’ normal care and to their involvement in the care organised/provided by mental health care professionals.

In addition to the supporting factors mentioned by patients, most relatives added that they ensured that concrete issues, like doing laundry or other practical support, were addressed. We found that parents were more often involved in arranging practical matters and caring than partners, even when their children were adults. In reply to why they helped, relatives noted that it made sense to do so because they wanted the patient -their family member- to recover. Some relatives also noted that they functioned as a “back-up” for patients. For instance, some patients lived with their parents for a while, because they could not live independently during a crisis.

N6: *“And she lived at home for a while*,* and then*,* especially with her and my wife*,* at a certain point they began to get on each other’s nerves. Then it had to go her way*,* the house was no longer ours*,* that’s how it seemed. So at a certain point*,* yeah*,* it got more and more irritating. Then we said*,* you really have to live on your own again.”*

Several professionals encouraged relatives to contact them if the patient was not doing well. In this way, the professionals remained informed. According to various professionals, relatives often have different interests than patients, because they have different perspectives on the situation. For this reason, some professionals suggested that it is sometimes better not to involve relatives. However, most professionals stated that they also needed relatives to keep the mental health care system running. For instance, relatives can take patients for a walk; professionals do not have time for such activities, which are important for a patient’s recovery.

H1: *“Because this is actually a kind of strategic issue that in a few years*,* but already now*,* there is a staff shortage. We will have to ask for more informal help; otherwise care won’t be tenable.”*

Finally, most professionals emphasised that it is important for patients also to have contact with non-professionals like their relatives because a stable social network is relevant for the patient’s recovery.

H4: *“I think: for treatment you need a practitioner*,* but for recovery and just moving on … just a person’s actual social development*,* really moving on and breaking free from just being sick… it’s good just to start exercising at Basic Fit and making contacts there.”*

#### Changing roles from relative to caregiver

Most patients mentioned they did not want to change their relationship with their relatives into a patient-caregiver relationship. For some, this was a reason not to involve relatives in the triad.

P2: *“In my case*,* they are my friends. And then*,* every so often*,* there are talks. I didn’t want that*,* I found it annoying*,* I found it stupid. They’re my friends*,* not caregivers.”*

The role of relatives thus often changed into that of a caregiver. We found that partners and parents differed with respect to this changing role. Partners often tended to adopt a more detached perspective than parents. Furthermore, the dynamic between partners and patients shifted, while still maintaining a relatively equitable balance. When the partner with mental health problems had more or less recovered, the normal pattern returned.

R1: *“Well*,* kind of a wing he can shelter under when it’s necessary.”*

We also noted that parents remained parents and kept caring, even though their child was an adult, although normally, parents take more distance from their children when they become adults. One professional indicated that she sometimes encouraged parents to resume their parental role rather than be disguised caregivers.

The sibling we interviewed noted that her role changed to being a caregiver when her sibling was not able to care for himself because of his mental illness.

#### Relatives’ intermediary role in patient-professional relationship

Both relatives and professionals noted that relatives have an intermediary role in the triad. Relatives perceived themselves as intermediates between professionals and their family members. They mentioned joining the patient at meetings with health care professionals, as well as making notes and asking questions during these meetings. When a patient, for example, could not speak coherently, relatives could understand what the patient meant and transfer this information to the professional.

R7: *“So for her*,* it was extremely important that I went along so that I could always translate. That helped*,* that helped my wife. Of course*,* it also helped me*,* but I think it also helped the practitioner to assess the situation correctly.”*

Professionals also regarded relatives as “their eyes and ears”. Whereas professionals see their patients once or twice a week, relatives see the patient more often. As a result, all relatives mentioned providing information regarding patients during the time between the meetings with professionals. For example, they could describe the side-effects of new medication, or provide other relevant information.

H3: *“So they are a bit like your eyes and ears as caregiver*,* because you are not always there*,* especially in the first stage. You can’t see everything and be everywhere.”*

#### Negative experiences of relatives in the triad

Many relatives pointed out that they were unsatisfied with their position in the triad. Most wanted to be more involved, especially during moments when the situation of their family member was deteriorating. Some said that to be part of the triad, they sometimes had to be assertive. As a result, relatives regularly felt unheard and unseen by professionals. We noted that men and women reacted differently to this situation. Male participants expressed more frustration, whereas women described more anxiety.

R5 (male): *“They have to help the boy to talk. They need to do more for the boy*,* get on his back. But if he doesn’t want to*,* say the supervisors*,* then it won’t happen. And so*,* I repeat*,* we don’t get a single step further.”*

Relatives mentioned having a small social network to share their worries with. Furthermore, they noted that they did not always want to discuss the situation of their child or partner because they did not expect it to change. They felt ashamed, or afraid of stigmatization.

R2: *“So you keep it to yourself*,* and you can’t share it with those close to you*,* your loved ones*,* because in my case*,* the ones who wouldn’t be judgmental have vulnerabilities of their own*,* or their child does*,* so you don’t want to bother them. So that was the end of it. Or they weren’t there anymore*,* like my mother.”*

As indicated by our research, that most patients have only a small social network means that they do not have many people to involve in the triad and are dependent on their relatives.

R3: *“Because they try hard to stimulate him to make contact with you…They also have a schedule for it*,* but that network has become very small. That is us*,* an aunt*,* a friend. And we are all in the same village*,* that’s about it.”*

Several relatives mentioned that they sometimes wanted the patient to be more independent in order not always to be needed themselves.

#### Ambivalence about patient’s autonomy

Some patients reported a lack of autonomy in deciding whether to involve relatives. Several patients expressed no desire to involve their relatives, either because they did not wish to burden them or preferred to manage independently. For some, involving relatives even felt obligatory.

P1: *“I don’t necessarily need it. But X and Y both say: let’s continue with the support group. We think it is good*,* and it is useful for you*,* they said. Now*,* I think things are going pretty well with me lately.”*

Additionally, several professionals in our study indicated that, in theory, patients should determine the composition of their support group (formal and informal networks). However, in practice, this is often not the case.

H3.2: *“In practice*,* the professional determines who belongs in the informal and formal network as patients are not always able to deal with this because of psychological problems*,* particularly in crisis situations.”*

Finally, most professionals mentioned that they experienced struggles between providing care and respecting patients’ autonomy. This struggle sometimes defines the ambivalence about patient autonomy. Their struggle was about the responsibility they felt between knowing what was best for the patient and the patient’s own direction and wishes.

## Discussion

This study examined collaboration within the triad, encompassing patients, relatives, and professionals, in a recovery-oriented context. As we explored various perspectives on this collaboration, we identified five themes: *i*) *the significance of involving relatives*,* ii changing roles from relative to caregiver*,* iii) relatives’ intermediary role in patient-professional relationship*,* iv) negative experiences of relatives In the triad*,* and v) ambivalence about patient’s autonomy*. These themes have a clear impact on triad collaboration. Notably, our study revealed an imbalance in collaboration among all participants.

### Themes within the triad

The first theme explores the *significance of involving relatives* in the recovery process. Participants highlighted several aspects of this involvement. According to patients, the significance of relatives is expressed in the opportunity to share experiences, offer stability, and collaborate on concrete issues. These roles align well with the mechanisms for recovery proposed by several scholars [34–37]. These mechanisms encompass moral, emotional, and practical support. We observed the following similarities: sharing experiences is a form of moral support, stability underlies emotional support, and practical support ensures practical problem-solving. However, it is worth noting that some patients choose not to involve their relatives, despite recognizing the positive effects of family involvement. From their perspective, despite the burdens, relatives expressed love and care for their family. This is also recognised in the literature, professionals endorsed the importance of family involvement [38]. They underlined that relatives help to maintain the system and that a healthy support group contributes to a patient’s recovery [35, 39, 40]. Nowadays, methods combining informal and formal networks, such as resource groups, are also gaining prominence, as underscored by various authors [41, 42].

Participation in the triad influences the role of relatives, i.e., they have to deal with *changing roles from relative to caregiver*. According to patients, the most important negative effect is family members transforming into caregivers. This affects their relationship with their relatives. This concern has also been noted in other studies, for example the tension between different roles, in this case role oriented problem solving and more focused on maintaining normal contact [43–45]. Relatives encountered “ambivalent relationships”, becoming disguised caregivers instead of parents or partners, which can cause them stress [9, 28, 46]. This stress arising from one’s role(s) in life may be defined as a situation whereby an individual finds himself unable to satisfy the expectations and commitments associated with a variety of roles [47]. These feelings may be linked to the theme of negative experiences. In this respect, our research indicated a difference between partners/siblings and parents. Partners and siblings regained their role of partners or siblings when their spouse or sibling recovered. Parents, however, kept worrying whether their children could again have a good and independent life. This is confirmed in other studies [39, 48, 49]. Interestingly, most of these studies focus more on parents than on partners. In line with recent literature, we also noticed that the role of relatives in recovery-orientated care was changing from caring as a normal task of life to a more official kind of care, explicitly relied on by professionals; relatives as their “eyes and ears” [38, 50].

Our study showed that relatives’ role in the triad changed over time. One of these roles is the *relatives’ intermediary role in patient-professional relationship*. Relatives act as intermediaries between patients and professionals. They actively try to collaborate with health care providers, striving to influence decisions. However, they are not readily granted such collaboration; to be allowed in the triad they must be assertive. One explanation for this is that the relationship between health professionals and patients is increasing, prompted by the field of shared decision-making (SDM), the availability of medical information, and the accessibility of electronic patient files. SDM aims to empower patients and families by involving them in informed decisions about treatment plans. It involves understanding patient wishes, managing expectations, and sharing relevant information [51–54]. With this increasing collaboration between patients and professionals, the relative often seems to be overlooked. Hence, one can speculate, as stated by Greenfield and colleagues, whether the collaboration is becoming more of a dyad than a triad [55]. Our research revealed that almost every relative is functioning as an intermediate. Surprisingly, in the literature, this role is hardly explored extensively as a separate research subject [4, 27]. Thus, we propose that Twiggs’ model, which is still in use, to be extended to include this intermediate role [3].

The *negative experiences of relatives in the triad* are related to their role in the triad. Our study found that relatives often desired greater involvement in the care process. When this involvement failed to materialise, they were reported to have feelings of despair or helplessness. This is not a new finding [8, 56, 57]. The article by Burger et al. elaborates on the experience of struggle [45]. In the present study, some relatives observed that professionals avoided involving them in treatment decisions. They expressed a sense of not being heard or seen by professionals. This contrasts with professionals’ observation that relatives are important for patients’ recovery as well as being important in other ways, such as being the professional’s eyes and ears [36]. This observation aligns with findings from other articles, emphasizing the importance of taking relatives’ needs seriously and fostering effective communication between relatives and professionals [58]. Presumably, a dismissive attitude on the part of professionals may be because they often find that patients and relatives have different interests [59]. For example, patients are reluctant to take medication, whereas the family insists on its necessity. Another example is patients sometimes want to take risks (e.g. stop medication) while family members are very keen for the situation to remain stable and not change too much.

Interestingly, in our relatively small study, male relatives reacted differently than their female counterparts, tending to feel frustrated rather than worried. However, the literature rarely describes the male perspective; only two studies highlight a gap in understanding male caregivers’ experiences [46, 50]. It is evident that the data about the before mentioned subject is limited in scope; consequently, it is essential to exercise caution when drawing conclusions of far-reaching nature.

Notably, our data revealed that relatives often have limited social networks, and it can be argued that this causes enormous stress [60]. This can also be seen as a restricted social network [61]. So far, little research has been done specifically on this topic, compared to research on patient networks in general. Furthermore, a broad body of literature underscores the vital role played by social networks in patient recovery [16, 36, 62, 63]. At the same time, relatives reported that patients often have small networks [44, 64, 65]. This lack of a supportive network renders patients more dependent on professionals and on their relatives within the triad, subsequently leading to more negative experiences for relatives as caregivers [56, 60].

Some patients expressed a strong sense of lack of autonomy in the triad. This contrasted with what was said by professionals, who advocated for patient autonomy, which makes sense because a lack of (social) autonomy negatively affects the recovery process, as argued by Bergamin [66]. We encountered a high level of *ambivalence* on the subject *of patient’s autonomy.* Some patients do not feel free to choose not to involve their relatives, either because some do not have a good relationship with their relatives, or they do not want to bother them [55]. This also can reflect a matter of ethical challenge of confidentiality [67]. Other patients do not have this experience and like the fact that their relatives are involved in treatment. Zooming in on the role of professionals, we see that they struggle with the balance between their own professional responsibilities and the patient’s autonomy. Depending on the situation, they tend towards one side or the other. Several factors augment this struggle, the most important being the shift towards patient-centred interventions, which emphasise patient autonomy. Consequently, professionals are seeking a new balance between their own responsibilities and their respect for the role of the patient [37, 42].

To conclude, all themes described in this paper have a degree of interdependence.

### The uneven triad

#### Triangle and triad

**Fig. 1:**
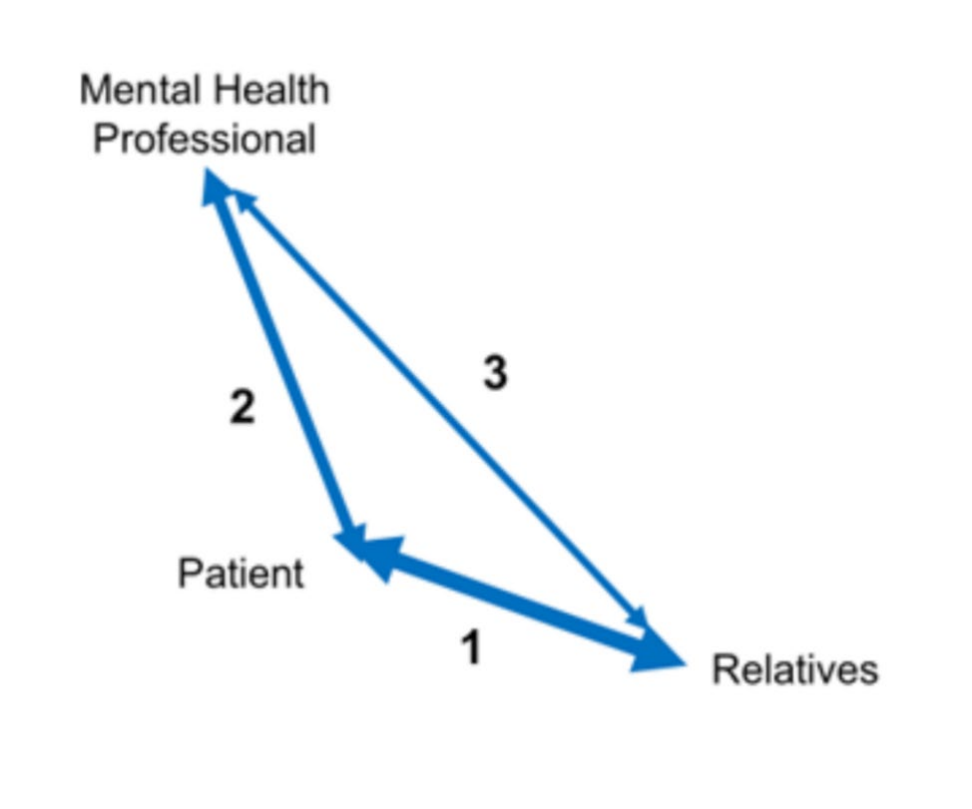
Collaboration in the triad, visualised

Collaboration in the triad is often depicted as a triangle with equal sides. However, as our data demonstrate, a triangle with uneven sides is a more realistic representation (see Fig. 1). Usually, the sides are connected to each other, but sometimes they are interrupted or non-existent. Therefore, we propose to speak of an “uneven triad”, a term not previously used in other papers.

Based on our study, the triad (see Fig. 11) would depict the distance between patient and relatives as the smallest ***(line 1)***, indicating they have the closest relationship. Next, the distance between a professional and a patient involved in a treatment relationship ***(line 2)*** is smaller than the distance between professional and relatives ***(line 3).*** This line 2 is a relationship with fairly clear boundaries. The boundaries between professionals and relatives are more ambiguous. According to our research, this resulted in a weaker relationship.

It is clear that the triad and its representation are highly dependent on a person’s actual situation. All relationships are dynamic, and each triad differs with different participants and -importantly- can change over time.

#### Tensions

Collaboration in the triad comes with tensions. Tensions between collaborating professionals and relatives are also described in the literature, especially when it comes to the influence both wish to exert on the course of the recovery process, their competing on who knows what is best for the patient [15]. These can be seen as diverging perspectives [45]. Another important tension that emerges from the literature and was also reported in our study is relatives’ feeling that they are not seen and heard by professionals, while the same professionals do acknowledge the importance of relatives in “keeping the system running“ [8, 22]. It is crucial that professionals become more aware of such tensions. To accurately describe the above issues, Worthington introduced a “disconnected model of involvement”, where relatives and professionals are not collaborating [68]. Our study showed that relatives do collaborate with professionals, albeit not without tensions. These tensions cumulate when relatives are seen mostly as resources, e.g., they were needed to keep the system running. This instrumental use of caregivers is also addressed by others [27, 69]. Based on our data, we see relatives becoming part of the system in their intermediary role. We may see this as a formalisation of their role, leading to additional tensions.

### Implications

Considering our search for new perspectives on involving relatives in recovery-based approaches, we can formulate several implications for *practice.* Various authors suggest strategies to improve the relationship between professionals and relatives. According to the literature, the health professional would do well not to be defensive, but to be open to comment, to be aware of what the relatives are going through, to involve them in the treatment, to offer encouragement, and not to be condescending [10, 14, 57, 63]. These important observations were also put forward by our participants. Tensions related to family involvement should be understood and validated by professionals, especially since professionals consider family members important for patients’ recovery [36, 57, 70]. There are many recovery-based approaches, e.g., the Active Recovery Triad-Model among others, which can help to shape the collaboration with relatives [17]. Further, the actual involvement of family members in shared decision-making can be supportive not only for themselves, but also for the patients and the professionals. Over the last decades mental health care has focused on patients as the centre of care. Now, it is highly necessary to focus (more) on the relatives as well. Even more so, considering the relatives’ emotional struggles reported above.

For *further research* on collaboration within the triad, we suggest examining the various tensions among all perspectives and elucidating ways to ease the tensions, such as among relatives who are not feeling heard or seen. We also recommend the study of the relative’s role as intermediary. The impact of an ill relative on the relationship in the triad requires serious attention because it may render collaboration less effective. As relatives are considered important, this deserves more attention in future research. Finally, we propose a study to enhance our scarce knowledge about male relatives and also on the small social networks of relatives.

### Strength and limitations

For our study we interviewed 24 participants, from all three parts of the triad: relatives, patients, and professionals. However, we were not able to include actual triads, i.e. relatives, patient and professional from the same triad. While it is not possible to state with certainty that data saturation can be achieved, we have carefully investigated this issue and attempted to achieve data saturation. It is possible that the experience of the participants was influenced by the ongoing pandemic of COVID-19. These might be limitations of this research. One of the strengths of this study is that we included all three perspectives through the lens of collaboration, whereas previous studies mostly concentrated on the relationship between patients and professionals only [39, 44]. The following observations can be made with regard to the trustworthiness of the study. We think we can account for the credibility, dependability, confirmability and transferability of it [71]. Firstly, the sample size is not large, but with enough variation. Secondly, member checks were carried out, analyses were conducted with several researchers (triangulation of researchers), and triangulation of perspectives. The first author had prior acquaintance with some of the interviewees, due to her involvement in other professional capacities. Through bracketing and team discussions, the issue of potential bias was addressed during the course of the discussion.

## Conclusion

Using a qualitative approach, our study uncovers varying perspectives on involving relatives and on collaboration within the triad. Recovery-based health care emphasises the participation of relatives., however these approaches do not always align with patients’ and relatives’ intentions and values. Limited social networks, as reported by relatives and patients, can arguably intensify relatives’ emotional struggles and therefore needs more attention. Relatives’ roles -especially as intermediaries- are surprisingly underexposed in the recovery-oriented literature. This study reveals several tensions among all perspectives, culminating in an uneven triad. To conclude, although involving relatives is highly advocated in recovery-based approaches, the practice is lagging behind. Until this changes, the triad will remain uneven.

## Data Availability

The datasets generated and/or analysed during the current study are not publicly available due to participant privacy; however, they are available from the corresponding author upon reasonable request.

## Declarations

The study was approved by the Medical Ethical Review Board of the University Medical Centre Groningen (201900607). Our study was performed by the declaration of Helsinki. Prior to the interviews, all participants provided written informed consent.

## Abbreviations

SMI: Severe mental illnesses
SDM: Shared decision making
F: Female
M: Male
SK: Suzanne Kroon
MA: Manna Alma
LvdK: Lian van der Krieke
RB: Richard Bruggeman
